# Awareness of testicular cancer among adult Polish men and their tendency for prophylactic self-examination: conclusions from Movember 2020 event

**DOI:** 10.1186/s12894-022-01098-1

**Published:** 2022-09-12

**Authors:** Jakub Ryszawy, Maksymilian Kowalik, Jakub Wojnarowicz, Grzegorz Rempega, Michał Kępiński, Bartłomiej Burzyński, Paweł Rajwa, Andrzej Paradysz, Piotr Bryniarski

**Affiliations:** 1 Division of Medical Sciences in Zabrze, Department of Urology, Medical University of Silesia in Katowice, Katowice, Poland; 2 Department of Rehabilitation, Faculty of Health Sciences, Medical University of Silesia in Katowice, Katowice, Poland; 3 Department of Urology, Medical University of Vienna, Vienna, Austria

**Keywords:** Health education, Health attitudes, Male, Self-examination, Surveys and questionnaires, Testicular neoplasms, Urology

## Abstract

**Background:**

Testicular cancer (TC), due to its non-specific symptoms and occurrence in young men, is particularly dangerous. A critical point for early diagnosis is awareness of the disease and the willingness to perform a testicular self-examination (TSE). The main aim of the study was to assess the knowledge of 771 adult men about testicular cancer. Additionally, the sources of information on TC and TSE were analyzed and the influence of demographic factors on the willingness to join preventative programs was examined.

**Materials and methods:**

The study was carried out during the Movember2020 campaign, where a testicular ultrasound was performed on participants. They were asked to complete a questionnaire with 26 questions to assess their knowledge.

**Results:**

The results obtained in the study indicate a low level of knowledge (average 3.5 points out of 18) about TC. Living in a large city (OR = 1.467; *p* = 0.03), as well as an earlier conversation about TC (OR = 1.639; *p* = 0.002), increased the awareness about the disease. Additionally it showed that many participants do not perform TSE at all (52.4%) and that only few perform TSE frequently (18.4%). Relationship status (OR = 2.832; *p* < 0.001) and previous conversations about TC (OR = 1.546; *p* = 0.02) was reported to be the main contributing factors in males deciding to have TSE.

**Conclusions:**

Our research indicates large educational neglect in terms of knowledge about TC and reluctance in performing TSE. It is worth carrying out preventative actions periodically on an increasing scale, not only for the screening of testicular cancer, but also to expand knowledge on this subject.

**Supplementary Information:**

The online version contains supplementary material available at 10.1186/s12894-022-01098-1.

## Background

Testicular cancer (TC) is a rare neoplasm occurring in men in Poland, as it represent only 1.6% of male malignancies. However, looking at the incidence rate in relation to the age structure, it is the most common cancer in the 20–44 age group and accounts for as much as 25% of all malignant neoplasms [1]. Epidemiological studies also show that the incidence is clearly higher in Central and North-Western Europe, thus compared to other regions of the world, the prevalence there is 5–10 times higher [2, 3]. Worldwide, TC is diagnosed in over 50,000 cases per year (data from 2018) compared to other more common urological cancers such as prostate, kidney or bladder cancer, which in 2020 were 191,930; 45,520; 62,100 cases respectively in the United States itself [4].

From the histopathological point of view, most of the testicular carcinomas are not epithelial in origin, but are derived in 95% from germ cells [5]. Despite its pathological origin being different, the name “testicular cancer” was adopted and is commonly used nowadays. Testicular tumours derived from germ cells can be classified as follows: seminoma (52% of tumours) and nonseminoma (48%). In the vast majority of cases, pure seminoma tumours dominate in the 4th decade of life [6].

Currently, none of the scientific societies recommends screening for TC [6–8]. Testicular self-examination (TSE) is the most basic testing method available. However, we observe a lack of randomized trials determining the frequency of its implementation in order to make it effective and reliable [9]. The European guidelines state that TSE should be performed among young patients with a high risk of developing TC [10]. The risk factors for this cancer are known, therefore to identify a group of men burdened with the disease is undemanding. These risk factors that may predispose to develop testicular cancer include:

Caucasian ethnicity (the risk of developing cancer is 4 to 5 times higher than in other ethnicities) [1].

Age (the National Cancer Registry lists the peak incidence between 20 and 44 years of age) [1, 11].

Cryptorchidism (failure of the testicle / testes to enter the scrotum, the increased risk persists despite possible surgery to bring the testicle into the scrotum) [1, 12].

Tumour in the second testicle (increases the risk by about 5%) [5, 13].

Patient height (there are several studies confirming a directly proportional relationship between height and the risk of testicular malignancy) [13].

Other (fertility disorders, hypocrisy, Klinefelter's syndrome, Down's syndrome, HIV) [1, 13].

Scientific research does not confirm the increased risk of cancer formation in patients with injuries or inflammation of the testicles. The increased detectability of tumours in this case most likely results from more frequent imaging diagnostics in the form of scrotal ultrasound. Also, no relationship was found between the increased temperature of the seat and the development of testicular cancer [1, 13, 14].

It is believed that there are hitherto unknown environmental factors that significantly increase the risk of TC. These conclusions result from the fact that the number of cases not related to the previously known disease factors is still growing [15, 16].

The most common symptom of TC with which the patient reports to the doctor is a hard, painless, palpable lump. Pain is a much less common symptom. It can result from a tumour haemorrhage caused by aggressive growth. Some patients report a problem related to non-specific discomfort and a feeling of heaviness in the scrotum [17].

Awareness of young men who are particularly exposed to TC is still at a very low level [11, 18]. Both in Poland and in the world, Movember campaigns are organized, which are primarily aimed at fast TC diagnostics by performing an ultrasound examination of the testicles. Another important aspect of this action is raising awareness about testicular cancer and its risk factors through information leaflets and the opportunity to talk to the doctor performing the test. The history of Movember's shares in Poland dates back to 2014 [19]. During the Movember 2020, an attempt was made to summarize the current state of knowledge about TC and to assess the frequency of TSE among young Poles taking part in the event.

The main aim of the study was to assess the knowledge of men on testicular cancer and the risks associated with this disease. Participants’ source of information regarding TC and contributing factors in undergoing self-examination and screening were analyzed. The final goal of the study was to analyze the demographic factors influencing the decision to enter testicular cancer prevention programs.

## Materials and methods

### Study design

Our study was conducted during the Movember campaign from October to December 2020 in Poland. The data was collected from 771 male participants, who filled out an anonymous questionnaire voluntarily. The study-specific questionnaire consisted of 26 questions, 22 were closed-ended and 4 could be answered with more than one answer.

The campaign dates were made public and each of the patients who applied for the study was asked to complete an anonymous questionnaire. Patients completed questionnaires before entering the doctor's office, where physical and ultrasound examinations of the testicles were performed. The survey was created for the purposes for our research.

### Statistical analysis

Categorical variables were analyzed with Chi-square test. Quantitative variables had non normal distribution as assessed by Shapiro- Wilk test. All these distributions were left skewed. To show the differences between quantitative variables, the U-Mann–Whitney test (comparison of 2 groups) or the Kruskal–Wallis test (when more than two groups were compared) were used. Additionally a multivariate logistic regression analysis was used to determine the influence of independent factors on knowledge about TC and the frequency of self-examination. Model for this analysis was constructed by backward elimination. *P* values < 0.05 were adopted as statistically significant. Statistical analysis was performed using the Statistica 13.3 program (TIBCO Software, CA, USA) (Additional files 3, 4 and 5).

### Consent of the bioethics committee

The study was prospectively submitted to Bioethics Committee of the Medical University of Silesia in Katowice, and concluded that the survey conducted does not require the consent of the Bioethics Committee on October 23, 2020.

## Results

### Analysis of the study group in terms of demographics and sociology

771 men took part in the study. Their average age was 30.6 years. Almost half of the respondents (49.3%) lived in cities with more than 500,000. residents. 27.4% of the respondents live in 100–500 thousand cities, 8.6% were inhabitants of 50–100 thousand, 8.5% were inhabitants of 50–10 thousand, and 6.1% were inhabitants of less than 10 thousand. These data show that residents of large cities are more willing to submit to screening tests. However, it should be taken into account that in large cities there is more access to information, including information about the Movember campaign. Therefore, there is a risk that such a low turnout of inhabitants of small towns may be related not only to the reluctance to take part, but also to the insufficient scale of spreading the Movember campaign.

The respondents with higher education constituted the largest group (67.5%). 31.5% had secondary education and 1.0% primary education. The dominant character of work among the respondents is intellectual work—66.3%. 14.2% were physically employed. There were 17.4% of pupils and students.

Another criterion that was assessed is whether the examined person is in a relationship (regardless of the status—formal or not). Men who were not in a relationship at all constitute 21.7%. The remaining respondents were in relationships, the largest group of which were those staying in a relationship for over 5 years (39.8%).

The frequency of sexual activity was as follows: as much as 49.3% of respondents had sex 1–4 times per week; 1–4 times per month was reported by 30.8%, men sexually inactive constituted 13.5%, and finally 6.3% of men were sexually active every day.

The most common reason for taking part in the campaign was prophylactic examinations (60.5%). The second reason was concern for health (11.6%) and 11.5% of patients accidentally came to the examination.

### Analysis of knowledge about testicular cancer

Questions 17–20 (Additional file 1) were intended to test the knowledge of the risk factors, symptoms and epidemiology of testicular cancer. Additionally, questions 17 and 18 were multiple-choice which containing both normal and abnormal risk factors and symptoms of TC. The question about TC risk factors contained 14 selectable answers, half of which were incorrect. When asked about the symptoms of the disease, out of 11 answers, 9 were correct and 2 were incorrect. Positive points were awarded for correctly indicating each correct answer, while for selecting the wrong ones (indicating ignorance) negative points were awarded. Introducing negative points to the total score was necessary due to the fact that the respondents could select all possible answers and despite the lack of knowledge, they would have to receive positive points for correctly indicating the correct risk factors or symptoms.Failure to mark the wrong answers (despite the fact that it would allow for a better verification of the respondents' knowledge) was not scored, due to the need to award points to the respondents who would leave the question unfinished. Question 17 verified knowledge of testicular cancer risk factors and was the biggest problem for the respondents (Tables 1, 2).

**Table 1:** Percentage of respondents indicating correct answers

Risk factor	Percentage of respondents (%)
Cryptorchidism	21
Ethnicity	5
Cancer in a first-degree relative	74
Fertility disorders	23
Genetic defects	58
Testicular cancer in the past and in the other testicle	46
High stature	2

**Table 2:** Percentage of respondents indicating incorrect answers

Risk factor	Percentage of respondents (%)
Carrying the phone in the pocket	49
High body weight	17
Alcohol	27
Increased temperature (e.g. laptop held on lap, heated seats)	44
High-fat diet	11
Previous vasectomy	8
Testicular injury	40

**Fig. 1:**
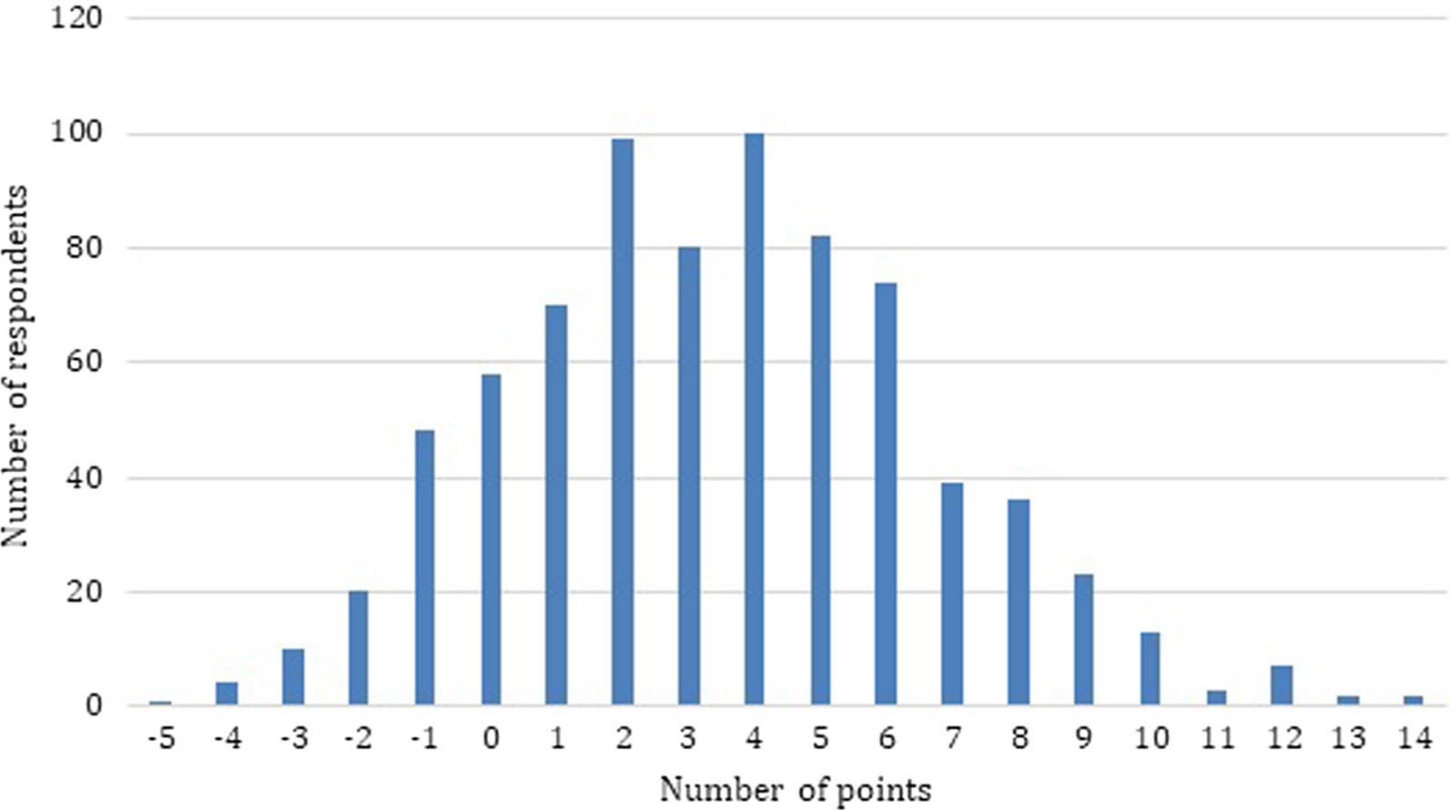
Number of points obtained by respondents in the knowledge test

**Fig. 2:**
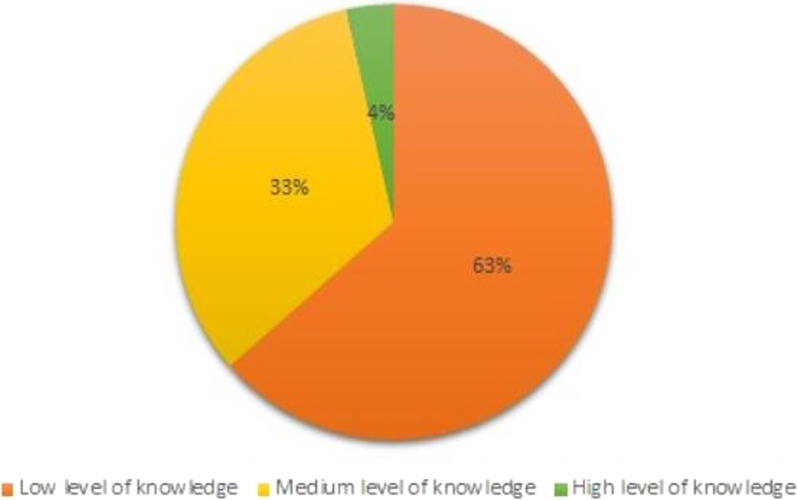
The state of knowledge of the studied group about TC

Questions 19 and 20 were single-choice questions and for selecting the correct answer 1 point was obtained for each of them. All points obtained by the respondents (both positive and negative) were summed up. In order to summarize the state of knowledge of the respondents, an (original) rating scale was created and the results obtained by the respondents were compared with it. This scale made it possible to divide the study group into people with low, medium and high level of knowledge (Table 3). As a result, the scoring range for questions 17–20 was from − 9 to 18 points (Fig. 1). The lowest score obtained was − 5 points. The highest score was 14 points and it was obtained by 2 men. The average was 3.5 points. The percentage distribution of the group depending on the level of knowledge is presented in Fig. 2.

**Table 3:** Original scoring scale assessing men's knowledge about testicular cancer

Number of points scored	Level of knowledge
≤ 4 points	Low
5–9 points	Medium
≥ 10 points	High

**Fig. 3:**
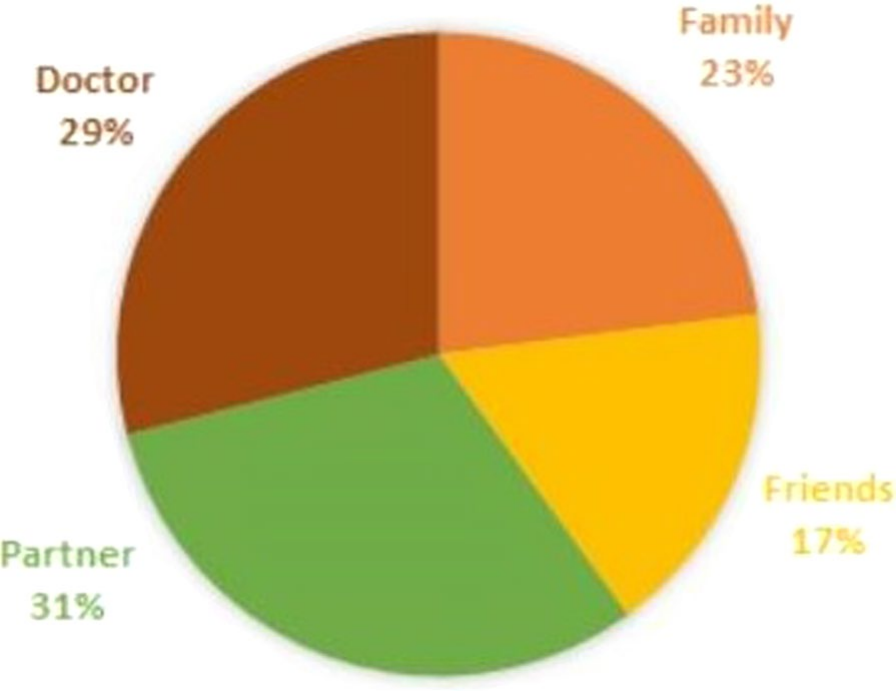
Percentage distribution of interviewees about testicular cancer. Most of the respondents (64.6%) did not talk about testicular cancer at all. The remaining respondents, 35.4%, showed that they start a conversation on this topic, and Fig. 1 shows who they talk to

Another important question included in the survey was whether the respondent was talking to someone about TC. If the person replied in the affirmative, they then had to indicate who they spoke with (Fig. 3). The full picture obtained in the conducted study is presented in Table 4.

**Table 4:** Responses of the respondents to selected closed questions asked in the survey

Question	Answer the respondent to the question asked yes (%)
Has any of your relatives had testicular cancer?	3.6
Has your friend had testicular cancer	16.1
Did you do a testicular ultrasound in the previous Movember action?	8.2
Will you attend next year?	90.7

**Fig. 4:**
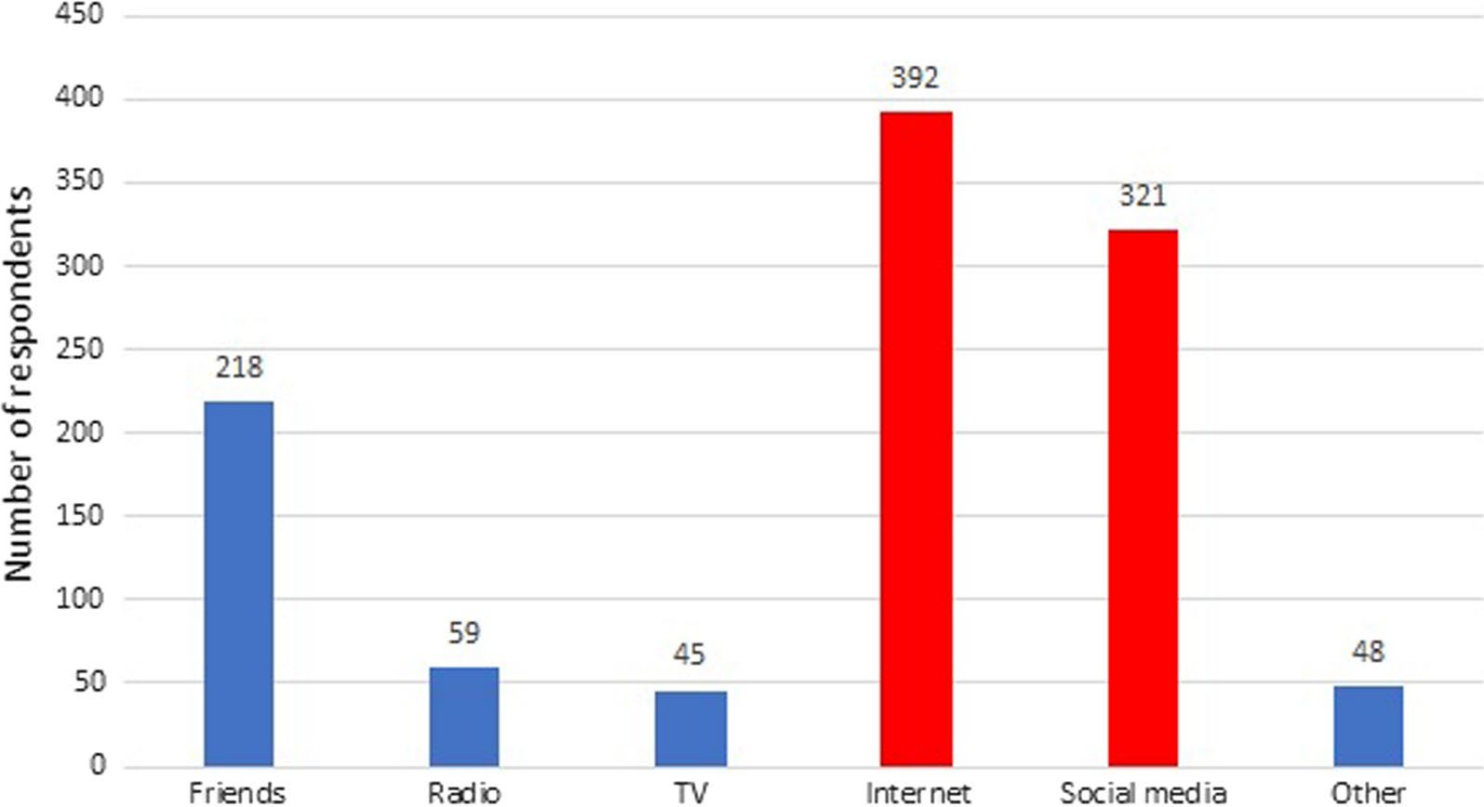
Sources of knowledge on testicular cancer

Another important issue we examined was the source of knowledge from which respondents learned about the testicular cancer campaigns (Fig. 4). It was observed that the main sources of knowledge about the action were the Internet (51%) and social media (42%). Among social media, Facebook was indicated the most often.

### Knowledge about testicular self-examination and the frequency of its performance

**Fig. 5:**
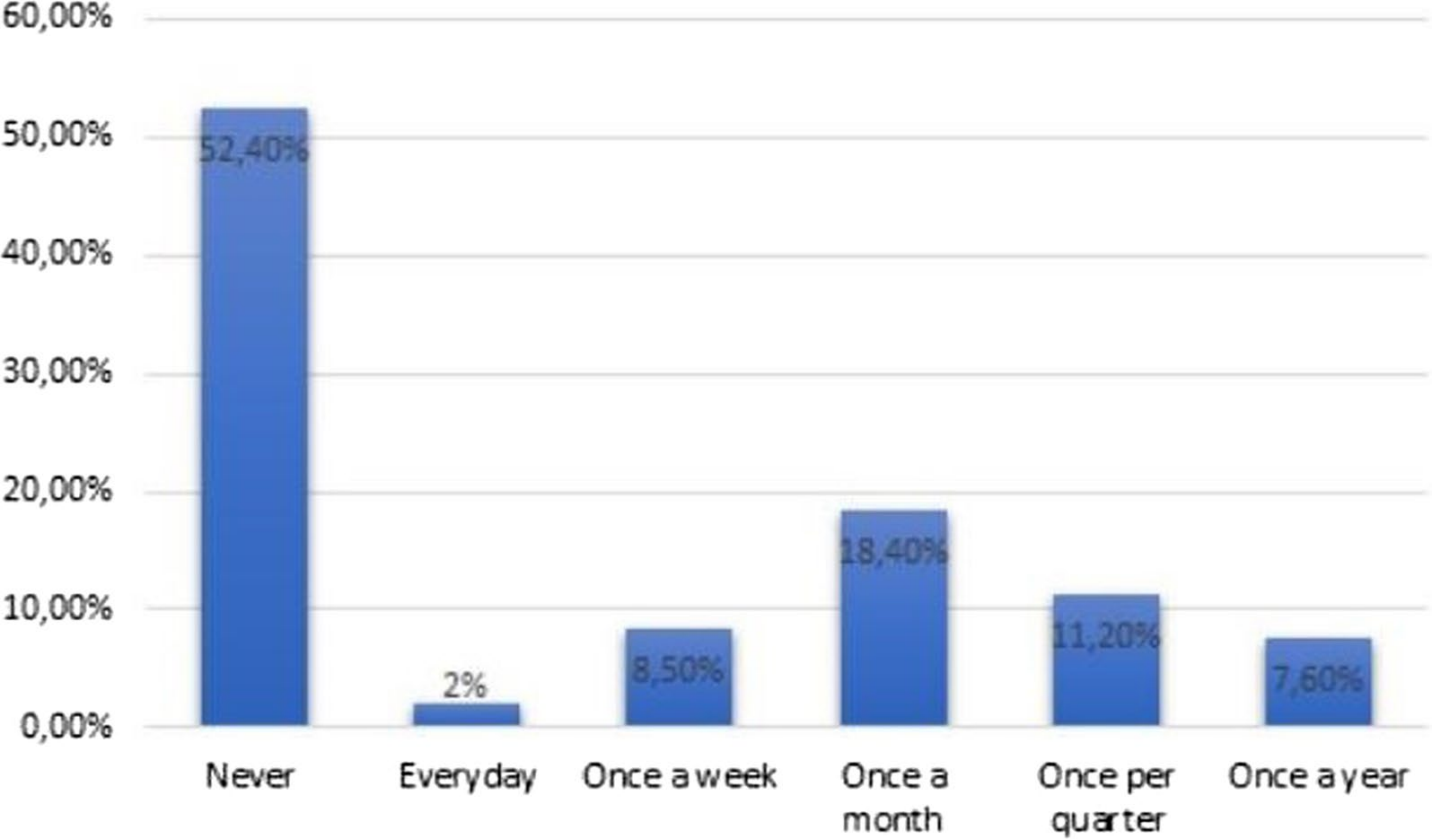
Frequency of TSE execution

The vast majority of respondents (77.6%), had heard about testicular self-examination. Respondents who were aware of TSE then had to indicate how often they performed them (Fig. 5). Of all the respondents, 46% correctly indicated the frequency (once a month) with which the testes should be tested according to the guidelines.

### Factors influencing knowledge about TC and willingness to perform TSE

**Fig. 6:**
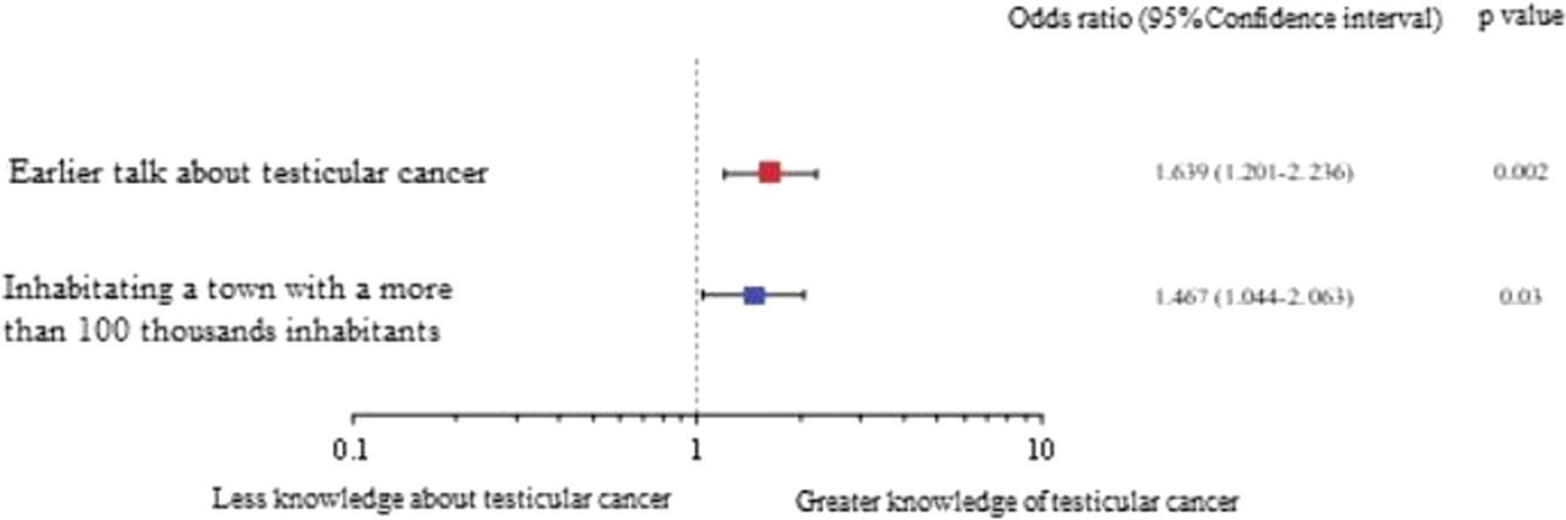
Influence of selected factors on the level of knowledge about TC

The multivariate statistical analysis showed which factors most significantly influenced the state of knowledge about TC. This analysis unequivocally indicated that both the inhabitance of a city of over 100,000 residents (OR = 1.467; *p* = 0.03), and an earlier conversation about TC, e.g. with a doctor, or partner (OR = 1.639; *p* = 0.002), significantly increases the level of knowledge about TC (Fig. 6). Other variables in model 1 including occupation, sexual activity, being in relationship were not significant (Tables 1–3 in Additional file 2).

**Fig. 7:**
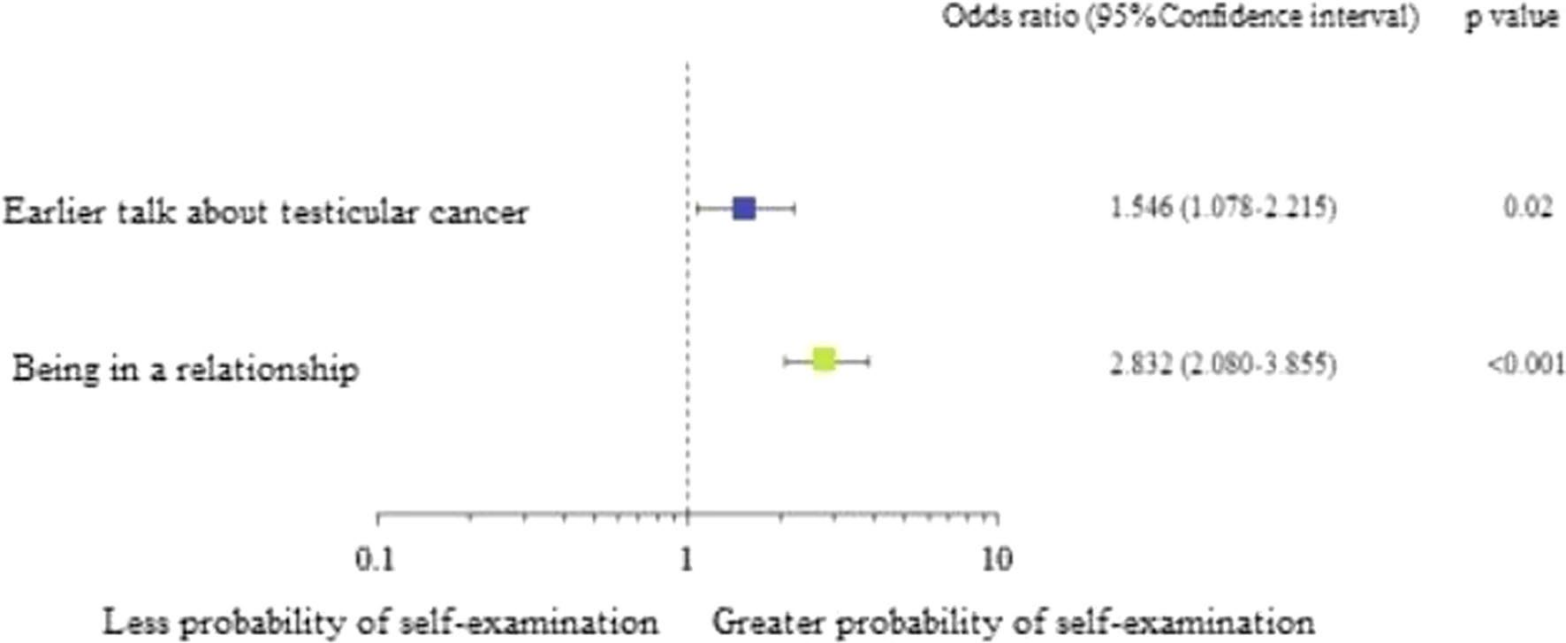
Influence of selected factors that increase in probability of TSE

Another result from the statistical analysis indicated factors that increase the probability of TSE. Being in a relationship (OR = 2.832; *p* < 0.001) and an earlier conversation about TC, e.g. with a doctor, or partner (OR = 1.546; *p* = 0.02) had a significant impact on increasing its frequency (Fig. 7).

Other variables in the model 2 including occupation, sexual activity, age, inhabitant of the city of over 100,000 residents were not significant (Table 4–6 in Additional file 2).

Goodness of fit was calculated with Pseudo R—it was 0.6 and 0.62 in model 1 and 2 respectively (Tables 2 and 5 in Additional file 2).

## Discussion

One of the primary aims of this study was to assess the awareness of potential testicular cancer risk factors. The knowledge about TSE and factors which prompt men for TSE were also analyzed.

The survey showed that 63.5% of the respondents had a low level of knowledge about testicular cancer. The study group was assessed as highly representative, as the mean age was 30.6 years—age most often affected by this type of cancer. The result obtained in the study indicated a very low awareness of men and a large educational neglect in this subject.

In another study, also carried out in Poland, researchers analyzed the level of knowledge of university students and interestingly, found that their understanding of TC risk factors is low as well. The results of this experiment are in-line with the results of our research. For example, cryptorchidism was indicated as a risk factor by only 20% of high school students and 38% of medical students. For comparison, in our study this answer was indicated by 21% of respondents. In addition, many people indicated incorrectly risk factors, e.g. the use of heated seats by 26.1% of students and 22.4% of high school students. The same answer was given by 44% of the men in our study. Summing up, the authors stated that medical students significantly more often indicated correct answers, which their field education certainly influences [11].

A similar study was conducted in Germany on students of medicine and other faculties. It showed that the level of knowledge about TC among students of the medical faculty was higher than among students of other faculties. This is analogical with the conclusions obtained by Polish researchers [20].

The countries located in Central Europe are mostly developed countries, however, according to research, there is a big concern about appropriate level of knowledge and awareness about TC [21–23].

It has been shown that being a resident of a large city with over 100,000 has a significant impact on increasing knowledge. This is most likely due to many factors, such as the possibility of taking advantage of various information events. Preventive campaigns, such as Movember, are organized in larger cities to increase awareness and knowledge about TC. The Movember action in many countries resulted in an increase in information about TC [24]. An important effect of our survey was the assurance that the respondents were willing to participate in the Movember campaign next year. This gives a good chance that some of the respondents will undertake a follow-up ultrasound examination of the testicles in the following years, and thus there is a greater chance of timely detection of TC and its appropriate treatment.

Additionally, on the topic of testicular self-examination, many (77.6%) respondents have merely heard of it. However, it is worth asking yourself whether the mere awareness of the possibility of TSE actually mobilized these patients to perform it. Unfortunately, our research shows that most men, despite the knowledge about self-examination (52.4%), do not perform it at all.

Other Polish scientists have also reached similar conclusions [25].The authors show that 57.5% of Polish men do not perform TSE at all. Among the group of people who perform the test: 12% do it once a month, 5% once a quarter, and 26% once every six months. Comparing these results with those obtained by us, it can be assumed that they are similar.

Though, it is worth noting, that the study [11] shows a much higher percentage of men not performing TSE compared to other analyses. The paper reports that > 80% of high school students and > 50% of medical students do not perform testes self-examination. However, when it comes to the frequency of taking TSE, the authors conclude that medical students choose to take it more often than high school students, and 30% of them did it at least once a month.

Whether or not the man was in a relationship was the factor that played a role in making the decision to start performing TSE. Previous conversations with a partner or a doctor also had an influence. As you can see, it is very important to raise the topic of TC in a relationship, as this conversation may persuade a man to undertake testicular self-examination. Other researchers also drew similar conclusions [22], stating that the partner's concern increases the likelihood of TSE performance.

Other, less frequently indicated factors were: family history of TC, recommendation of a general practitioner (GP) and social campaigns. The paper [25] emphasizes the importance of discussion with GP about TC, therefore teaching TSE by a doctor increases the chances that the patient will perform it. A patient instructed by a doctor has more confidence that he/she performs the tests correctly.

All the works included emphasize the role of the physician in building awareness of testicular cancer. They also show how essential and valuable it can be to pass on knowledge about TSE by the family doctor with whom the patient has the greatest contact among other specialists.

Another conclusion that was obtained in the studies [26–28] is the fact that the increased frequency of TSE performance improves its effectiveness. With each subsequent examination, the patient is more precise and vigilant, thus there is a greater probability of noticing disturbing changes in the testicles.

Unfortunately, even men who are aware of TC and self-examination do not want to discuss it with other people. It is still viewed as a personal issue and something to be shy of. The data contained in the work from 2019 indicate that over 50% of men aware of self-examination never shared their knowledge, even with friends [29]. This is why it is important to educate people about testicular cancer and TSE in the early stages of school education and then continue in secondary and university education [30–33]. In particular, education should focus on the knowledge of risk factors, as only 5% of the respondents correctly indicated ethnic origin as a factor predisposing to TC. Similarly, only 2% indicated high stature as a risk factor. Also, cryptorchidism, which is the main risk factor, was correctly marked by only 21%. Education should also clearly indicate that carrying a phone in a trouser pocket is not a factor predisposing to TC, as this answer was incorrectly indicated by almost 50% of respondents.

For comparison, it is worth paying attention to how education, social campaigns and screening mammographs influenced the early diagnosis of breast cancer and the promotion of self-examination among women. It used to be an embarrassing topic, and today the vast majority of women are aware of the usefulness of self-examination and are able to perform it. A similar effect can be achieved in testicular cancer. Teaching youth self-examination may increase their involvement in preventive health care in adulthood [34, 35].

One of the limitations of our study is the lower availability of information about health campaigns or access to a doctor among residents of smaller towns. Undoubtedly, this fact influenced the results of our work.

## Conclusions

The results of the study indicate a low level of men's knowledge about TC. In order to make a change, it is worth paying attention to the school curriculum. Routine implementation of testicular cancer education can raise youth awareness. Increasing the knowledge about the risk factors and symptoms of TC may help speed up the detection of neoplasms.

Also, science and the promotion of TSE are crucial elements that can affect the faster detection of the disease. Physicians, especially those of first contact, also play an important role in both screening components.

Promotional campaigns such as Movember are a very useful tool for disseminating knowledge about TC and TSE due to their reach. Supporting such activities on social media (next to the Internet indicated by the reporter as the most common source of knowledge) will certainly bring significant results in terms of increasing knowledge about testicular cancer.

## Supplementary Information

**Additional file 1:** Questionnaire.

**Additional file 2:** Logistic regression analysis.

**Additional file 3:** Descriptive characteristics.

**Additional file 4:** Reasons for taking TSE.

**Additional file 5:** Self-examination table.

## Data Availability

The datasets used and/or analysed during the current study available from the corresponding author on reasonable request.
