# Correction to: Awareness of testicular cancer among adult Polish men and their tendency for prophylactic self-examination: conclusions from Movember 2020 event

**DOI:** 10.1186/s12894-023-01189-7

**Published:** 2023-02-26

**Authors:** Jakub Ryszawy, Maksymilian Kowalik, Jakub Wojnarowicz, Grzegorz Rempega, Michał Kępiński, Bartłomiej Burzyński, Paweł Rajwa, Andrzej Paradysz, Piotr Bryniarski

**Affiliations:** 1 Division of Medical Sciences in Zabrze, Department of Urology, Medical University of Silesia in Katowice, Katowice, Poland; 2 Department of Rehabilitation, Faculty of Health Sciences, Medical University of Silesia in Katowice, Katowice, Poland; 3 Department of Urology, Medical University of Vienna, Vienna, Austria

**Correction : BMC Urology (2022) 22:149** 10.1186/s12894-022-01098-1

Following publication of the original article [1], the authors would like to update the affiliation information of co-author Dr. Pawel Rajwa.

It has been updated in this correction and the original article has been corrected.
