# Supplementary material for: Awareness of testicular cancer among adult Polish men and their tendency for prophylactic self-examination: conclusions from Movember 2020 event
Source: BMC Urol. 2022 Sep 12;22:149. doi: 10.1186/s12894-022-01098-1 (PMC9469579; doi:10.1186/s12894-022-01098-1)
Supplement: Supplementary file 1 — Additional file 1: Questionnaire. [file 12894_2022_1098_MOESM1_ESM.docx]

Title : Testicular Cancer Knowledge Questionnaire

Legend: Survey It contained 26 questions, of which 22 were closed questions, and 4 could be given more than one answer.

Knowledge about testicular cancer

**1. Initials**

**2. Age**

**3. Place of residence [thousands]**

0-10

10-50

50-100

100-500

Over 500

**4. Education**

Primary

Secondary

Higher

**5. Profession**

White-collar worker

Manual worker

Pupil / student

Pensioner / annuitant

Unemployed

**6. Are you in a relationship? If so, how long?**

Not

Less than a year

1-5 years

Over 5 years

**7. Height [cm]**

**8. Body weight [kg]**

**9. Native language**

Polish

English

German

Spanish

Other:

**10. Why did you come for the examination?**

Something bothered me

My partner sent me

Prophylactically

My company organized the examination

Accidentally

Other:

**11. Have you had ultrasound examinations carried out in previous years as part of Movember?**

Yes

No

**12. Will you take part in the action next year?**

Yes

No

**13. Did any of your first degree relatives (father, brother, son) have testicles cancer / tumor?**

Yes

No

**14. Has your friend had a testicular tumor / cancer?**

Yes

No

**15. How did you hear about the testicular cancer campaign? [select all correct]**

friends

radio

TV

Internet

social media

poster

Other:

**16. If you found out about the action from social media, select from which**

Facebook

Instagram

Twitter

Other:

**17. Tick ALL the risk factors for testicular cancer you know:**

*Tick all that apply.*

wearing the phone in pants

cryptorchidism (undescended testicle, testicle outside the scrotum)

race (skin color)

large body weight

alcohol

cancer in a first-degree relative

fertility disorders

increased temperature (laptop on lap, heated car seats)

genetic defects

the presence of a tumor in the second testicle in the past

high-fat diet

high hight

previous vasectomy (ligation of the vas deferens)

testicular trauma

**18. Tick ALL the symptoms of testicular cancer you know**

*Tick all that apply.*

palpable tumor

aching testicles

testicular enlargement

gynecomastia (nipple enlargement and pain in men)

change in testicle consistency / cohesiveness

burning sensation when urinating

back / abdominal / groin pain

feeling of heaviness in the scrotum

swelling of the scrotum

premature puberty

premature ejaculation

**19. What is the age range of testicular cancer incidence?**

0-14

15-45

46-65

Over 65

**20. Which place in terms of prevalence in young men among all of malignant tumors is testicular** **cancer?**

First

2-5.

5-9.

Except the first 10

**21 Have you ever spoken to anyone about your testicles (distressing symptoms)?**

Yes

No

**22. . If so, with whom (select all correct ones)**

*Tick all that apply.*

family member

colleague / friend

partner

health care representative (doctor, nurse)

Other:

**23. Testing of the testicles. Have you heard about self-examination?**

Yes

No

**24. . Testing of the testicles. Do you test yourself, if so, how often?**

I'm not testing myself

every day

weekly

once a month

once per quarter

once a year

**25. Testing of the testicles. How often SHOULD I be tested?**

every day

weekly

once a month

once per quarter

once a year

when I feel something

**26. Are you a sexually active person? If so, how often do you have sex?**

I am not sexually active

every day

1-4 per week

1-4 per month
