# Supplementary material for: Awareness of testicular cancer among adult Polish men and their tendency for prophylactic self-examination: conclusions from Movember 2020 event
Source: BMC Urol. 2022 Sep 12;22:149. doi: 10.1186/s12894-022-01098-1 (PMC9469579; doi:10.1186/s12894-022-01098-1)
Supplement: Supplementary file 2 — Additional file 2: Logistic regression analysis. [file 12894_2022_1098_MOESM2_ESM.docx]

|  | Odds ratio (95%CI) | Wald statistics | P value |
| --- | --- | --- | --- |
| Having talked to someone about testicular cancer (yes/no) | 1,639 (1,201-2,236) | 10,61 | 0,002 |
| Living in a city >100000 (yes/no) | 1,467 (1,044-2,063) | 5,33 | 0,03 |

Table1. Modeling for probability of high knowledge of testicular camcer with significant variables only. Reference levels: no.

|  | Predicted no | Predicted yes |
| --- | --- | --- |
| Observed no | 396 | 91 |
| Observed yes | 209 | 70 |

Table 2. Observed vs predicted in model 1. Pseudo R square = 466/766 = 0,6

|  | Odds ratio (95%CI) | Wald statistics | P value |
| --- | --- | --- | --- |
| Age | 0,99 (0,97-1,01) | 0,007 | 0,92 |
| Relationship (yes/no) | 1,12 (0,76-1,65) | 0,33 | 0,56 |
| Having talked to someopne about testicular cancer (yes/no) | 1,68 (1,23-2,3) | 11,02 | <0,01 |
| Living in a city >100000 (yes/no) | 1,35 (0,93-1,97) | 2,53 | 0,11 |
| Occupation (white collar worker/other) | 1,32 (0,93-1,86) | 2,55 | 0,10 |
| Sexually active (yes/no) | 0,97(0,62-1,51) | 0,06 | 0,89 |

Table 3. Modeling for probability of high knowledge of testicular cancer with all variables. Reference levels: No, white collar worker.

|  | Odds ratio (95% CI) | Wald statistics | P value |
| --- | --- | --- | --- |
| Beeing in relationship (yes/no) | 2,832 (2,080-3,855) | 44,36 | <0,001 |
| Talking to someone about testicular cancer (yes/no) | 1,546 (1,078-2,215) | 5,47 | 0,02 |

Table 4. Modeling for probability of self examination with significant only variables. Reference levels: No.

|  | Predicted no | Predicted yes |
| --- | --- | --- |
| Observed no | 303 | 98 |
| Observed yes | 191 | 174 |

Table 5. Observed vs predicted in model 2. Pseudo R square = 477/766 = 0,62

|  | Odds ratio (95%CI) | Wald statistics | P value |
| --- | --- | --- | --- |
| Age | 0,99 (0,97-1,01) | 0,008 | 0,92 |
| Relationship (yes/no) | 1,54 (1,04-2,26) | 4,86 | 0,02 |
| Having talked to someopne about testicular cancer (yes/no) | 2,88 (2,11-3,94) | 44,16 | <0,01 |
| Living in a city >100000 (yes/no) | 0,79 (0,55-1,13) | 1,58 | 0,2 |
| Occupation (white collar worker/other) | 1,01(0,72-1,43) | 0,01 | 0,91 |
| Sexually active (yes/no) | 0,97(0,62-1,51) | 0,01 | 0,89 |

Table 6. Modeling for probability of self examination with all variables. Reference levels: No, white collar worker.
