# Supplementary material for: Awareness of testicular cancer among adult Polish men and their tendency for prophylactic self-examination: conclusions from Movember 2020 event
Source: BMC Urol. 2022 Sep 12;22:149. doi: 10.1186/s12894-022-01098-1 (PMC9469579; doi:10.1186/s12894-022-01098-1)
Supplement: Supplementary file 3 — Additional file 3: Descriptive characteristics. [file 12894_2022_1098_MOESM3_ESM.docx]

| Variable | Whole study group  n=771 |
| --- | --- |
| Age (years; median and quartile range) | Average=30.6±8.1; Median=30 (25-35) |
| Domicile |  |
| <10,000 residents | 6.1% |
| 10-50 thousand residents | 8.5% |
| 50-100 thousand residents | 8.6% |
| 100-500 thousand residents | 27.4% |
| > 500,000 residents | 49.3% |
| Education |  |
| Basic | 1.0% |
| Medium | 31.5% |
| Higher | 67.5% |
| Profession |  |
| Intellectual | 66.3% |
| Physical | 14.2% |
| Pupil / student | 17.4% |
| Pensioner / retiree | 0.7% |
| Unemployed | 1.4% |
| In a relationship |  |
| No | 21.7% |
| < 1 year | 10.1% |
| 1-5 years | 28.4% |
| >5 years | 39.8% |
| The reason for applying for the test |  |
| Worried about symptoms | 11.6% |
| Convince a partner | 9.9% |
| Prevention | 61.5% |
| Advertisement in the company | 1.0% |
| Accidentally | 11.5% |
| Other | 4.7% |
| Did you have an ultrasound scan in the previous Movember action? |  |
| Yes | 8.2% |
| No | 91.8% |
| Will you attend next year? |  |
| Yes | 90.7% |
| No | 9.3% |
| Family history of testicular cancer |  |
| Yes | 3.6% |
| No | 96.4% |
| Testicular cancer in a friend |  |
| Yes | 16.1% |
| No | 83.9% |
| The subject spoke to someone previously about testicular cancer |  |
| Yes | 35.4% |
| No | 64.6% |
| If yes, with who? |  |
| Faminy | 13.4% |
| Friend | 10.0% |
| Partner | 18.6% |
| Doctor | 17.0% |
| Are you sexually active? How often? |  |
| No | 13.5% |
| Everyday | 6.4% |
| 1-4 times a week | 49.3% |
| 1-4 times a month | 30.8% |
| Have you heard about self-examination? |  |
| Yes | 77.6% |
| No | 22.4% |
| Are you testing yourself? How often? |  |
| No | 52.4% |
| Everyday | 2.0 |
| Once a week | 8.5 |
| Once a month | 18.4% |
| Once per quarter | 11.2% |
| Once a year | 7.6% |
