# Supplementary material for: Awareness of testicular cancer among adult Polish men and their tendency for prophylactic self-examination: conclusions from Movember 2020 event
Source: BMC Urol. 2022 Sep 12;22:149. doi: 10.1186/s12894-022-01098-1 (PMC9469579; doi:10.1186/s12894-022-01098-1)
Supplement: Supplementary file 4 — Additional file 4: Reasons for taking TSE. [file 12894_2022_1098_MOESM4_ESM.docx]

| Variable | Because of partner | Upset | By accident | Another reason | p - value |
| --- | --- | --- | --- | --- | --- |
| Age (years; median and quartile  range) | 30 (27-34) | 29.5 (23-35) | 31 (24-37) | 29 (25-35) | 0.31 |
| Domicile |  |  |  |  | 0.78 |
| <10,000 residents | 5.26% | 8.05% | 5.75% | 5.87% |  |
| 10-50 thousand residents | 9.21% | 11.49% | 8.05% | 8.02% |  |
| 50-100 thousand residents | 9.21% | 12.64% | 5.75% | 8.22% |  |
| 100-500 thousand residents | 26.32% | 31.03% | 27.59% | 27.20% |  |
| > 500,000 residents | 50.00% | 36.78% | 52.87% | 50.68% |  |
| Education |  |  |  |  | 0.14 |
| Basic | 1.32% | 1.15% | 1.14% | 0.97% |  |
| Medium | 26.32% | 40.23% | 40.91% | 28.85% |  |
| Higher | 72.37% | 58.62% | 57.95% | 70.18% |  |
| Profession |  |  |  |  | 0.03 |
| Intellectual | 68.92% | 54.65% | 60.23% | 69.20% |  |
| Physical | 17.57% | 26.74% | 18.18% | 11.11% |  |
| Pupil / student | 13.51% | 17.44% | 18.18% | 17.35% |  |
| Pensioner / retiree | 0.00% | 1.16% | 1.14% | 0.58% |  |
| Unemployed | 0.00% | 0.00% | 2.27% | 1.75% |  |
| In a relationship |  |  |  |  | 0.004 |
| No | 3.95% | 24.14% | 25.00% | 22.96% |  |
| < 1 year | 9.21% | 16.09% | 9.09% | 9.34% |  |
| 1-5 years | 35.53% | 17.24% | 28.41% | 29.38% |  |
| >5 years | 51.32% | 42.53% | 37.50% | 38.33% |  |
