# Supplementary material for: Awareness of testicular cancer among adult Polish men and their tendency for prophylactic self-examination: conclusions from Movember 2020 event
Source: BMC Urol. 2022 Sep 12;22:149. doi: 10.1186/s12894-022-01098-1 (PMC9469579; doi:10.1186/s12894-022-01098-1)
Supplement: Supplementary file 5 — Additional file 5: Self-examination table. [file 12894_2022_1098_MOESM5_ESM.docx]

| Variable | Do not perform self-tests | Perform self-tests | p - value |
| --- | --- | --- | --- |
| Age (years; median and quartile range) | 30 (25-35) | 29 (25-35) | 0.97 |
| Domicile |  |  | 0.33 |
| <10,000 residents | 4.51% | 7.99% |  |
| 10-50 thousand residents | 8.52% | 8.26% |  |
| 50-100 thousand residents | 8.77% | 8.54% |  |
| 100-500 thousand residents | 26.82% | 28.10% |  |
| > 500,000 residents | 51.38% | 47.11% |  |
| Education |  |  | 0.29 |
| Basic | 0.50% | 1.65% |  |
| Medium | 31.92% | 31.04% |  |
| Higher | 67.58% | 67.31% |  |
| Profession |  |  | 0.52 |
| Intellectual | 67.17% | 65.56% |  |
| Physical | 13.28% | 15.15% |  |
| Pupil / student | 17.54% | 17.08% |  |
| Pensioner / retiree | 0.25% | 1.10% |  |
| Unemployed | 1.75% | 1.10% |  |
| In a relationship |  |  | 0.20 |
| No | 24.88% | 18.36% |  |
| < 1 year | 10.20% | 10.14% |  |
| 1-5 years | 26.87% | 30.14% |  |
| >5 years | 38.06% | 41.37% |  |
| The reason for applying for the test |  |  | 0.67 |
| Worried about symptoms | 11.06% | 11.81% |  |
| Convince a partner | 10.80% | 9.07% |  |
| Prevention | 61.31% | 61.81% |  |
| Advertisement in the company | 1.01% | 1.10% |  |
| Accidentally | 10.30% | 12.64% |  |
| Other | 5.53% | 3.57% |  |
| Participation in the previous Movember event |  |  | 0.98 |
| Yes | 8.23% | 8.26% |  |
| No | 91.77% | 91.74% |  |
| Family history of testicular cancer |  |  | 0 .52 |
| Yes | 3.24% | 4.12% |  |
| No | 96.76% | 95.88% |  |
| Testicular cancer in a friend |  |  | 0.27 |
| Yes | 17.46% | 14.56% |  |
| No | 82.54% | 85.44% |  |
| The subject spoke to someone previously about testicular cancer |  |  | <0.001 |
| Yes | 24.56% | 47.67% |  |
| No | 75.44% | 52.33% |  |
| The subject is sexually active |  |  | 0.64 |
| Yes | 85.93% | 87.09% |  |
| No | 14.07% | 12.91% |  |
| Testicular cancer knowledge (score; median and quartile range) | 3 (1-5) | 4 (2-6) | <0.001 |
